# Retraction: Knockdown of Tcf3 enhances the wound healing effect of bone marrow mesenchymal stem cells in rats

**DOI:** 10.1042/BSR-2018-0369_RET

**Published:** 2023-04-06

**Keywords:** bone marrow mesenchymal stem cells, rat, Tcf3, wound healing

This Retraction follows an Expression of Concern relating to this article previously published by Portland Press. This article is being retracted from *Bioscience Reports* at the request of the authors following receipt of a notification from a reader, alerting the Editorial Office to images that seem to appear in their paper and in papers by other, unrelated authors.

These include the following images:

- The Figure 2D Si-Tcf3#3 image, which seems to appear as the Figure 3C Inhibitor NC panel from Zhao et al. 2017 (doi: 10.1042/bsr20170837) and as the 143B/sh-VAV3+inhibitor panel of Figure 6E from Xiao et al. 2020 (doi: 10.18632/aging.103762)
- The Figure 2D Si-Tcf3#1 image, which seems to appear as the Figure 2C sh-NC panel of Bian 2020 (doi: 10.2147/OTT.S180850),
- The Figure 4G CK-18 and GAPDH western blots, which also seem to appear as the Figure 6A CaBP-9k bands and the 2C ER-beta bands respectively from Liu et al. 2020 (doi: 10.1042/bsr20191330).

The authors are unable to find the original data to correct the article and therefore wish to retract the article. The Editor-in-Chief and Editorial Board agree with the retraction.

